# Human endogenous retroviruses as immune-related prognostic biomarkers in pancreatic cancer

**DOI:** 10.1186/s12885-026-16373-9

**Published:** 2026-08-20

**Authors:** Paula Iuliana Toma, Laurentia Nicoleta Gales, Francois Ghiringhelli, Anda Trandafir, Raluca Ioana Budea, Silvia Mihaela Ilie

**Affiliations:** 1 University of Medicine and Pharmacy Carol Davila, Dionisie Lupu Street, No. 37, District 2, Bucharest, Romania; 2 Gral Medical Hospital, Medical Oncology Department, Bucharest, Romania; 3 Department of Medical Oncology, Institute of Oncology Alexandru Trestioreanu, Bucharest, Romania; 4 Personalized Medicine and Access to Therapeutic Innovations Unit, Georges Francois Leclerc Cancer Center, Dijon, France; 5 Cancer Biology Transfer Platform, Georges Francois Leclerc Cancer Center, Dijon, France; 6 Center for Translational and Molecular Medicine, INSERM UMR 1231, Dijon, France; 7 University Burgundy Europe, Dijon, 21000 France; 8 Department of Medical Oncology, Georges Francois Leclerc Cancer Center, Dijon, France

**Keywords:** Human endogenous retroviruses, Pancreatic ductal adenocarcinoma, Viral mimicry, Prognostic biomarkers, Systematic review

## Abstract

**Background:**

Human endogenous retroviruses (HERVs) may modulate antitumor immunity and prognosis in pancreatic cancer (PC), but reported findings remain heterogeneous.

**Methods:**

A systematic review was conducted according to PRISMA guidelines. PubMed/MEDLINE and grey literature were searched for studies reporting HERV expression in PC. Extracted data included HERV family and genomic region, detection methods, biological specimens, immune correlations, clinicopathologic associations, and survival outcomes.

**Results:**

Eleven studies were included, reporting 1,085 PC cases among 9,241 cancer patients. HERV-H was evaluated in 6 studies, HERV-K in 3, and 2 studies did not specify family. Elements included env sequences (3 studies), envelope proteins (2), LTR sequences (6), and LTR-derived proteins (HHLA2 (5). Detection methods comprised RNA-seq (*n* = 7 studies), PCR/qPCR (*n* = 7), immunohistochemistry (*n* = 6), western blot (*n* = 4), and ELISA (*n* = 4). HERV detection ranged from 16.7% to ~ 80%, varying with the sequence and laboratory method. Immune correlations were described in 7 studies, including associations with CD8⁺ T-cell infiltration, cytokines (IL-2, TNF-α, IFN-γ), PD-1/PD-L1, and anti-HERV antibodies. Overall survival was reported in 6 studies, with both adverse (HR 1.26–2.24) and favourable (HR 0.47–0.66) associations reported, particularly for HERV-H derived LTR products (HHLA2).

**Conclusions:**

HERVs exhibit frequent, target-dependent expression patterns that trigger proinflammatory activation. Within these families, HERV-H derived LTR elements, specifically HHLA2, demonstrate the most consistent correlation with increased T-cell infiltration and favorable survival, supporting their potential as prognostic biomarkers.

**Graphical abstract:**

**Figure:**
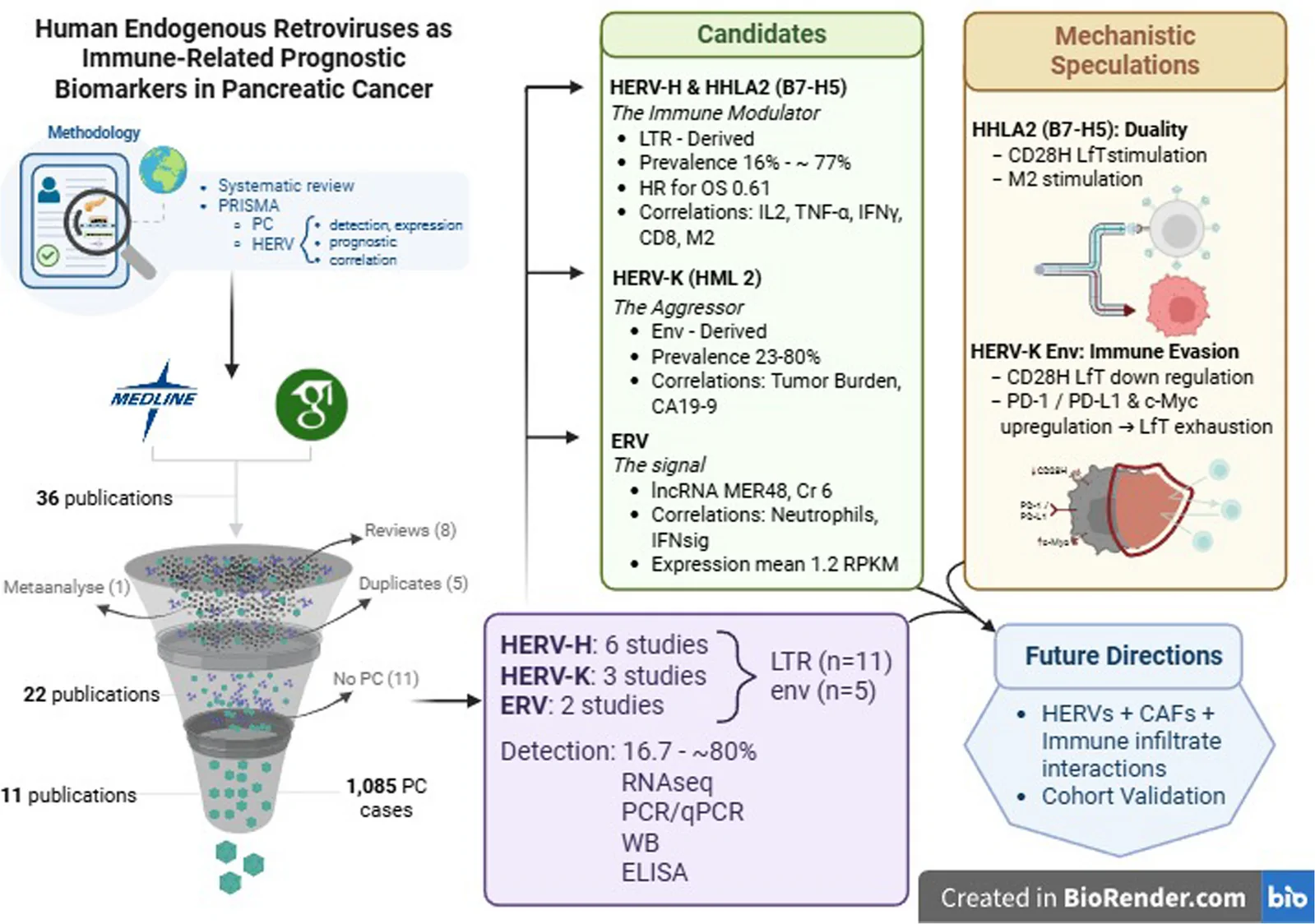

**Supplementary Information:**

The online version contains supplementary material available at 10.1186/s12885-026-16373-9.

## Introduction

Human endogenous retroviruses (HERVs) and HERV-like sequences represent 8% of the human genome and are transmitted in a Mendelian manner [1, 2]. Although long considered as non-functional deoxyribonucleic acid (DNA) [3], they are now recognised as contributors to genome regulation and innate immune signalling [4]. They can be reactivated epigenetically by chemical or physical agents, or viral infections [5]. Structurally, HERVs are composed of retroviral genes (gag, pro, pol and env) flanked by regulatory non-coding long terminal repeats (LTRs) [6]. Physiologically, HERV elements contribute to host immune responses to viral infection through viral mimicry [7], and to trophoblast development through reactivation of an env coding region [8]. However, their aberrant reactivation has been reported to be implicated in degenerative disease [9] as well as in oncogenesis [10].

Concerning immune-mediated mechanisms, reactivated HERVs primarily engage the innate immune system through double- and single-stranded RNA (dsRNA, ssRNA), complementary DNA (cDNA), and viral-like proteins, which are recognized by Pattern Recognition Receptors (PRRs). This recognition activates interferon regulatory factors, resulting in the production of type I interferons and pro-inflammatory cytokines, such as interleukins IL-1β and IL-18 [4]. Additionally, LTRs enhance transcription in response to IFN stimulation and induce an effective antiviral response [11].

Innate immune activation by HERVs can subsequently shape the adaptive immune response [12]. IFN signalling enhances antigen presentation by dendritic cells and macrophages, while env, gag and pol proteins activate CD8 + cytotoxic T cells [13].

Pancreatic ductal adenocarcinoma remains one of the most lethal malignancies, with limited therapeutic options and a five-year survival rate below 15% [14]. The biological aggressiveness of this disease is compounded by an immunosuppressive tumour microenvironment and a paucity of reliable prognostic and predictive biomarkers [15].

In pancreatic cancer, several studies have reported expression of distinct HERV families and genomic regions, including HERV-K and HERV-H–derived elements. The available evidence is heterogeneous because of diverse analytical platforms, biological materials and outcome measures, with inconsistent reporting of immune, clinical and prognostic associations [16–19].

We aimed to summarize the available evidence on HERV expression in pancreatic cancer, including HERV families, genomic regions and molecular products, their biological and immune correlates, and, where reported, their association with prognostic outcomes, with the goal of identifying candidate biomarkers of antitumor immunity.

## Methods

### Search strategy

In March 2025, we conducted an extensive search for publications in the PubMed/MEDLINE database and grey literature (Google Scholar) to identify specific expressed loci of human endogenous retroviruses (HERVs), a correlation between HERVs in pancreatic cancer and a potential prognostic role. Due to budgetary limitations, we were unable to include subscription-based databases such as Web of Science, EMBASE and Cochrane CENTRAL.

The search strategy was developed according to the PICO (Population, Intervention, Comparison, Outcome) framework and reported in compliance with the Preferred Reporting Items for Systematic Reviews and Meta-Analyses (PRISMA) guidelines [20]. Both free-text terms and Medical Subject Headings (MeSH) terms were used to capture all relevant studies. For the population (P), the research combined terms related to pancreatic adenocarcinoma; for the intervention (I), terms describing human endogenous retroviruses were included; for the comparison (C), terms referring to normal pancreatic tissue were added where applicable; and for outcomes (O), terms related to survival and prognosis were used. The literature search was structured using either phrases of controlled vocabulary or free-text keywords. Examples of the MeSH terms and free-text phrases used in the search strategy are provided below, and additional related terms and synonyms were applied to ensure comprehensive retrieval of relevant studies.

For the population, MESH terms included: “Pancreatic Neoplasms”, “Adenocarcinoma”, “Carcinoma, Pancreatic Ductal”, while representative free-text terms included pancreatic cancer, pancreatic adenocarcinoma, PDAC, pancreatic ductal adenocarcinoma, pancreatic carcinoma.

For the exposure of interest, MeSH terms included “Endogenous Retroviruses”, “Retroelements”, while examples of free terms are human endogenous retrovirus*, HERV*, HERV-K, HERV-H, HERV-W, HML-2, endogenous retroviral element*, ERV, LTR, long terminal repeat*, envelope protein, B7-H5, HHLA2.

The comparative component of the search was implemented using free-text terms only, as MeSH indexing did not permit adequate discrimination of non-malignant pancreatic comparator tissues, normal pancreatic tissue, adjacent normal tissue, healthy control*, normal pancreas*.

Outcome-related concepts were captured using MeSH terms, including: “Prognosis”, “Survival”, “Survival Rate” and free text terms such as survival, overall survival (OS), progression-free survival (PFS), disease-free survival (DFS).

Further details regarding the research methods are available in Supplementary File S1.

A manual review of the references cited in the identified articles was also performed to detect potentially relevant publications not retrieved in the initial search. Only publications in English were selected for inclusion.

### Study eligibility

In this analysis, we considered as eligible all articles that evaluated any HERV expression in pancreatic cancer, tissue, serum or TCGA, even if no association with the survival outcome was investigated.

We included observational studies (cohorts, case series), in vitro studies with clinical validation and in silico analyses that reported HERV expression of coding regions and/or non-coding regions or viral proteins. We investigated associations with clinical characteristics, such as tumour stage, histological grade, marker level, immune variables including tumour-infiltrating lymphocytes (TILs), cytokine levels and programmed death-ligand (PD-L1) expression.

Exclusion criteria were studies limited to in vitro or animal models, investigations focusing exclusively on non-pancreatic cancers, narrative reviews, editorials and meta-analyses.

Given the small number of included studies (*n* = 11) and the heterogeneous nature of study designs (in vitro experiments, in silico database analyses, clinical cohort studies and combined approaches with diverse outcomes), the Newcastle-Ottawa Scale (NOS) was applied to the observational cohort and case-series studies. The NOS evaluates three domains — Selection (maximum 4 stars), Comparability (maximum 2 stars), and Outcome (maximum 3 stars) — for a maximum total 9 stars; studies were graded as good (≥ 7 stars), fair (4–6 stars) or poor (≤ 3 stars). For the predominantly mechanistic in vitro studies and for the in silico TCGA/GTEx analyses, the NOS was considered not directly applicable; for these studies, a brief narrative appraisal of methodological strength was performed instead. The full per-study appraisal is provided in Supplementary File S2 and the findings inform the interpretation of results and are reflected in the limitations section.

### Data collection and analysis

Data extraction was performed independently by two reviewers, PIT and AT, using a preplanned data extraction form. Another author (RIB) compiled and entered the extracted data and the senior author (SMI) was the arbitrator in case of disagreements.

Data extracted from selected publications included study design, number of pancreatic cases among all tumour cases, the HERV family and genomic region analysed, including specific loci or molecular targets and reported outcomes. We also collected data regarding the detection method (e.g., transcriptomic, proteomic or serology), the biological material analysed, the type of comparator and the format of expression data (e.g., presence/absence, relative expression or absolute quantification). When available, we extracted data on the association between HERV expression and immune-related parameters as well as demographic or clinical pathologic variables. Prognostic endpoints, such as OS, PFS, DFS or equivalent, were systematically extracted and evaluated.

## Results

### Characteristics of included studies

**Fig. 1:**
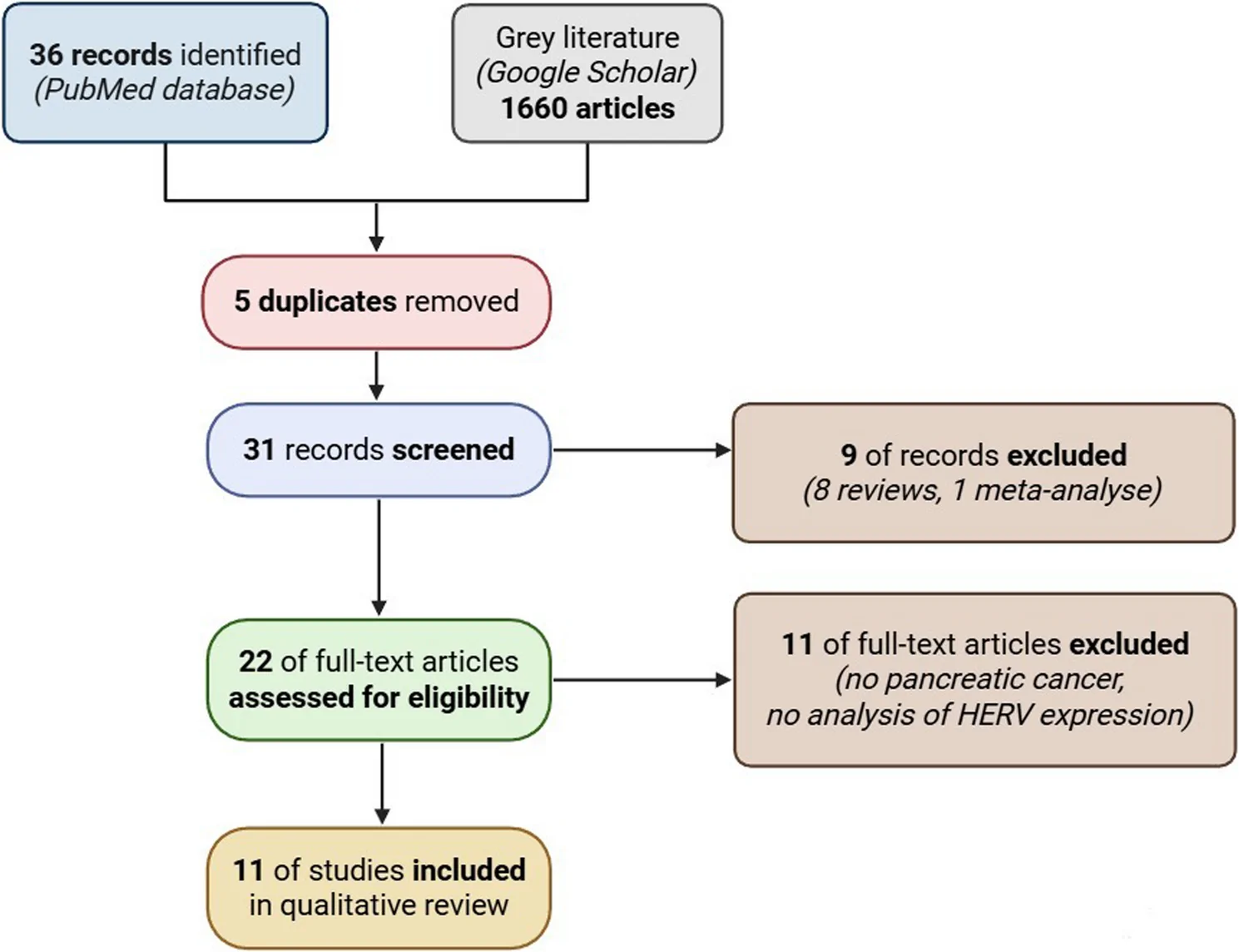
PRISMA Chart

We identified a total of 36 publications, including 5 duplicates, 8 reviews and 1 meta-analysis, leaving 22 articles for evaluation. Among these, eleven were excluded as being outside the scope of this review: seven investigated HERV expression in settings other than pancreatic cancer, two did not address pancreatic cancer, one evaluated the effect of Death-domain Associated protein X on HERV expression, and one evaluated the effect of MEK1/2 inhibition in pancreatic cancer (Fig. 1).

Among the eleven studies included a total of 1,085 patients with pancreatic adenocarcinoma were evaluated out of the initial cohort of 9241 cancer patients.

The publications comprised three case series [17, 21, 22], six exploratory cohort studies [16, 18, 19, 23–25] and two analysed exclusively public databases, namely The Cancer Genome Atlas (TCGA), The Encyclopedia of DNA Elements (ENCODE), Gene Expression Omnibus (GEO) [26] and Personalised OncoGenomics (POG), PanGen [27]. Four studies evaluated established pancreatic cancer cell lines [16, 21, 22, 26] and two combined analyses of in vivo clinical specimens with in vitro experimental models [17, 18].

Among the included studies, six assessed HERV-H [18, 19, 21–24], three evaluated HERV-K [17, 19, 25] and two reported ERV expressions without family specification [26, 27]. At the transcriptomic level, envelope(env) coding sequences were reported in three publications [17, 19, 21], whereas the corresponding encoded proteins were reported in two of them [16, 25]. Open reading frames (ORFs) were reported in two studies, one to the H family [21] and one to the K family [17]. The LTR sequences were reported in six studies [18, 19, 23, 24, 26, 27] and the protein (HHLA2/B7-H5), in five studies [18, 19, 22–24] (Table 1).

**Table 1:** Characteristics of included studies [1–27]

Nr.	Study (author/ year/ country)	Study design	Pancreas samples / Total cases	HERV family	HERV region	Outcomes a) Primary b) Secondary
I	Clinical cohort studies					
1.	Schmitz-Winnenthal FH, 2007, Germany [16]	Cell lines Clinical cohort	96/130	HERV-K	env	a) Detectability absolute expression b) Detectability absolute expression in normal tissue
2.	Chen Q, 2018, China [18]	Clinical cohort Cell lines Xenograft	136/136	HERV-H	B7-H5 (HHLA)	a) Inferential: IL 2, TNF-α, IFN-γ b) Detectability relative expression Inferential: T growth, CD28H Prognostic: OS
3.	Yan H, 2019, USA [23]	Clinical cohort Public database: TCGA	92/153	HERV-H	LTR-HHLA	a) Detectability relative expression b) Inferential: CD8T density. TNM, age, gender Prognostic: OS
4.	Boor PP, 2020, Netherlands [24]	Clinical cohort	122/194	HERV-H	HHLA	a) Absolute expression b) Prognostic on CSS, RFS Inferential: CA 19, R1/ R2, grade, ln, CD8^+^,FoxP3 T
5.	Zhou S, 2024, China [19]	Public database TCGA/ TIMER/ GTEx Clinical cohort Cell lines	345 62	HERV-H	HHLA	a) Relative expression prognostic OS b) Mechanistic: EGFR / MAPK / ERK, mTOR / AKT, M2
6.	Gong Q, 2025 China [25]	Clinical cohort Cell lines	100/400	HERV-K	env	a) Detectability absolute K102-Env level of expression Interferential TNM stage markers b) Mechanistic: ICI, cMyc oncogene
II	Case series					
7.	Wentzensen N, 2007, Germany [21]	Case series	2/12	HERV-H	ORF	a) Detectability relative expression b) Inferential: type of cancer LTR demethylation
8.	Byers JT, 2015, USA [22]	Case series Cell lines	15/23	HERV-H	B7-H5 (HHLA)	a) Detectability: relative expression b) Inferential: type of T tissue
9.	Li M, 2017, USA [17]	Case control Cell lines Xenograft models	30/30 106 (serum cohort)	HERV-K	Env Gag ORF (Rec NP9)	a) Relative expression b) Inferential: Serum Biomarkers: vRNA, Ab anti-HERV-K Mechanistic: RAS/ MEK /ERK, AKT, TP53, Novel splice variant
	In silico/ Public Database					
10.	Gibb EA, 2015, Canada [26]	Public database: TCGA ENCODE GEO Cell lines	52/7677	ERV 1	MER48-LTR	a) EVADR expression b) MER48 specificity EVADR Inferential: type of tissue, T stage Prognostic: OS
11.	Topham JT, 2020, Canada [27]	Public database POG PanGen	33 / 199	ERV	abundance of ERV-transcripts	a) Global Relative Expression b) Inferential: ERV transcripts, antiviral genes, DNA methylase TET2 Identification of a viral mimicry phenotype

*Abbreviations:* *POG* Personalized Oncogenomic, *PanGen* NCT 02869802 CT, *TCGA* The Cancer Genome Atlas Program, *ENCODE* The Encyclopedia of DNA Elements, *GEO* Gene Expression Omnibus, *HERV-K* Human endogenous retroviruses K family, *HERV-H* Human endogenous retroviruses H family, *HHLA* HERV–H LTR-associating 2, *K102-Env* HERV-K102 Envelope Protein, *ERV* Endogenous retrovirsus, *Env* Envelope glycoprotein, *LTR* Long terminal repeat sequences, *MER48* Medium Reiteration frequency repeat 48, *EVADR* Endogenous retroviral associated adenocarcinoma, *TIMER* Tumor Immune Estimation Resource, *R1 /R2* positive postsurgical margins, *ln* lymphnodes status, *M2* immunosuppressive macrophages, *T* Tumour, *TNM* Tumour, nodes, metastasis stage, *ORF* Open Reading Frame, *OS* Overall Survival, *Gag* Group-specific antigen, *Rec* Regulator of Expression, *NP9* Nuclear Protein 9, *vRNA* Viral Ribonucleic Acid, *Ab* Antibody, *RAS* Rat Sarcoma proto-oncogene, *MEK* Mitogen-Activated Protein Kinase, *ERK* Extracellular Signal-Regulated Kinase, *AKT* Protein Kinase B, *TP53* Tumor Protein p53, *IL* Interleukin, *TNF-α* Tumor Necrosis Factor alpha, *IFN-γ* Interferon gamma, *CD28H* Cluster of Differentiation 28 Homolog, *CD8* *+* CD8-positive cytotoxic T lymphocytes, *CD8T* CD8 T cells (cytotoxic T cells), *CSS* Cancer-Specific Survival, *RFS* Recurrence-Free Survival, *CA 19* Carbohydrate Antigen 19, *FoxP3* Forkhead Box P3, *DNA* Deoxyribonucleic Acid, *TET2* Ten-Eleven Translocation Methylcytosine Dioxygenase 2, *EGFR* Epidermal Growth Factor Receptor, *MAPK* Mitogen-Activated Protein Kinase signaling pathway, *ERK* Extracellular Signal-Regulated Kinase, *mTOR* Mechanistic Target of Rapamycin, *ICI* Immune Checkpoint Inhibitors, *cMyc* Cellular Myelocytomatosis Oncogene

### Detection methods

HERV expression was measured using multiple analytical platforms: RNA sequencing (7 studies), PCR/qPCR-based methods (7 studies), immunohistochemistry (6 studies), Western blot (4 studies) and ELISA or serologic assays (4 studies). These methods were applied to primary tumor tissue (11 studies), adjacent or normal pancreatic tissue (8 studies), serum samples (2 studies), cell lines (4 studies), public databases including TCGA and related resources (6 studies)(Table 2).

**Table 2:** Characteristics of detections methods and outcomes of HERVs sequences in pancreatic cancer [1–27]

Nr	HERV family	Specific locus / target	Analytical method	Measurement method	Biological specimen	Comparator	Expression outcome	Immune correlations	Demographic /clinical correlations	Prognostic endpoints
**1.**	HERV-K [16]	HERV-K-MEL	RT-PCR	Semi-quant	Primary tumor (surgery)	Non-matched normal tissues	23%	N/A	N/A	N/A
**2.**	HERV-K [17]	HERV-K (HML-2) env SU-K102-like, K113/ K115-like 794 bp variant	RT-PCR IHC RNA-Seq ELISA qPCR WB TEM	Qual Quant	Primary tumor (Biopsy) Cell lines Patient sera	Matched normal tissue Nonmalignant cell lines Normal donor sera	80% Present in 7 different PC cell lines	Anti-Env SU (*p* = 0.028) Anti-NP9 (*p* = 0.012) antibodies	Clinical stage: env (*p* = 0.004) Gag(*p* = 0.001)	N/A
**3.**	HERV-K (HML _2 subtype) [25]	K102 env K108 env	ELISA WB	Quant	Patient PAC Sera Jurkat T cell lines	Healthy sera K108env	63.33% K108-env 34% K 02-env (36.63 fold higher than in healthy controls)	PD-1 PD-L1 c-Myc	tumor type (68% sensitivity, 83% specificity), metastatic stage (*p* < 0.001), CA 19 − 9 (*p* < 0.01)	N/A
**4.**	HERV-H [21]	Xp22.3	RT-PCR NB Bisulfite Sequencing	Qual Quant	Primary tumor (surgery)	Matched Normal Tissue	≥ 3-fold: 16.7%	N/A	N/A	N/A
**5.**	HERV-H [22]	HHLA2 (LTR-derived protein)	IHC Flow Cytometry	Semi-Quant	Primary tumor (surgery)	Matched normal tissue	20%. adenocarcinoma *p* = 0.002 0% in cell lines	N/A	N/A	N/A
**6.**	HERV-H [18]	B7H5 (HHLA2)	Flow Cytometry IHC ELISA RT-qPCR	Semi-quant Quant	Primary tumor (surgery) Pancreatic cell lines	Panc-1 H149 cells Matched normal tissue	68.38% mRNA correlation (*p* = 0.0425)	Cytokines (IL-2, TNF-α, IFN-γ) T-cell proliferation	No association	OS week vs. strong Median: 11.5 vs. 16.5 months (*p* = 0.017) HR 2.241 (1.351–3.718), p 0.002
**7.**	HERV-H [23]	LTR/HHLA2	IHC	Qual Quant	Primary tumor (surgery)	Matched normal tissue	77.17%	CD8⁺ T density (*p* = 0.9194)	No association	Better OS (*p* = 0.0015)
**8.**	HERV-H [24]	HHLA2 (LTR-derived protein) HHLA2 mRNA primers	IHC RT-PCR	Semi-quant Quant	Primary tumor (surgery)	Healthy human tissues Ampullary cancer	67% Low: 49% High: 28% 100% mRNA correlation	CD8⁺ T (*p* = 0.006)	HHLA high T stage (0.042) anticorrelated CA19-9 (0.00) R1/R2 (0.012)	CSS: low vs. absent HR 0.601 (0.389–0.929) *p* = 0.022 High vs. low HR 0.471 (0.264–0.839) *p* = 0.011 Cancer Recurrence High vs. absent (*p* = 0.007)
**9.**	HERV-H [19]	HHLA2	Bioinformatic analysis IHC RT-qPCR WB IFS	Quant	Pancreatic cancer tissue Normal pancreatic tissue Pancreatic cancer cell lines	Matched normal tissue Negative control small interfering RNA	TCGA/GTEx: *p* < 0.0001 6-Paired Experimental Cases (WB and RT-qPCR) *p* < 0.01 62-Patient Clinical Validation Cohort (IHC) *p* < 0.05 Cell Line Comparisons (PC cells vs. Normal cells) *p* < 0.01	neutrophils and M2 macrophages M2 (*p* = 0.014) M1 (*p* = 0.0066)	No association	OS HR 1.256, (1.062–1.485), *p* < 0.008
**10.**	ERV [26]	MER48 LTR, chromosome 6	Bioinformatic RT-PCR Luciferase reporter assays 5’RACE	Quant	Primary tumor (surgery)	Matched Normal tissue	12.1 ± 17.2 RPKMS	N/A	N/A	Pan-adenocarcinoma analysis OS HR 1.47 (1.06–2.04) *p* = 0.02
**11.**	HERV [27]	ERV load	Bioinformatic *Differential* expression	Quant	Metastases (biopsy)	N/A(?)	High ERV only High antiviral only Low ERV VMP	IFN sig (*p* = 2.3 × 10^− 7^) T cells *p* = 6.9 × 10^− 3^ Neutrophils *P* = 4.6 × 10^− 5^ 72 genes antiviral score rho = 0.58 *p* = 0.0005	N/A	Log rank *p* = 0.63

One study by Li et al. performed laboratory-based RNA and DNA sequencing to assess the change in gene expression profile after knockdown of HERV K [17]. Bisulphite sequencing was used to determine the CpG methylation status of viral promoters [21].

Enzyme Linked Immunosorbent Assay (ELISA) provides absolute quantification of serum proteins, with one study establishing a detection limit of 0.03 ng/ml for HERV-K102 Env [25]. Other ELISA applications measured the concentrations of cytokines IL-2, TNF -alpha and IFN-gamma [18]. Levels of mRNA were assessed via reverse transcription polymerase chain reaction (RT-PCR) [16, 17, 21, 24] and quantitative real-time polymerase chain reaction (RT-qPCR) [18, 19]. Across the selected studies, 28 distinct bioinformatic tools were used for data processing, each serving a specific purpose: alignment (e.g. ultrafast universal RNA -seq aligner- STAR, Targeted Read Assembly -TASR, Basic local alignment search tool -BLAST etc.) [26], quantification (e.g. Cufflinks - transcript assembly and quantification, edge R-differential expression analysis) [17, 26], structural analysis (RepeatMasker-screens DNA sequences, MEGA-Molecular Evolutionary Genetics Analysis) [17, 27], pathway analysis (e.g. IPA – ingenuity pathways Analysis, CIBERSORT -cell type identification) [17, 19] and tools to evaluate coding potential [26].

Western blot was used to identify proteins such as p-ERK1/2 (extracellular signal-regulated kinase 1/2) [17], cMyc protooncogene derived [25] and EMT (epithelial-mesenchymal transition) [19]. Northern blotting was used to visualize the 5.4 kb HER-V sequence [21] and phosphokinase arrays to detect the relative phosphorylation of 43 kinases [17]. Cell behavior was assessed via Cell Counting Kit-8 (CCK − 8), colony formation (transformation), wound healing (migration) and transwell (invasion) assays [19]. Flow cytometry was used to assess T-cell proliferation using carboxyfluorescein succinimidyl ester (CFSE) staining and to monitor receptor expression, including CD28 homolog (CD28H) and programmed cell death protein 1 (PD-1) [22]. Dual-luciferase reporter assays were performed to evaluate the promoter activity of LTR elements [26]. In vivo research on pancreatic cancer within the provided sources utilised various immunodeficient mouse models to investigate the functional roles of retroviral markers like HERV-K and B7-H5 (HHLA2) in tumour growth and metastasis: nude and NOD/SCID mice, as in Li et al. [17] study or NSG and B-NSG mice as in Chen et al. [18].

### Overall expression findings

Reported levels of HERV-K env-derived elements varied across studies from 23% for HERV-K MEL transcript [16] to 38% for K102 protein, 63% for K108 protein [25] and up to 80% for HML-2 [17]. HERV-H env-specific locus located on chromosome Xp22.3, position 4.479.152–4.484.999, was found to be overexpressed in 16.7% of pancreatic cancers and 3-fold higher than in samples considered negative in the study by Wentzensen et al. [21]. Human Endogenous Retrovirus-H LTR-Associating (HHLA2), also known as B7-H5 expression, was found to be significantly upregulated in tumour versus adjacent normal tissue (*p* < 0.01) [19], strong staining was observed in 68.38% of cases [18] and 77.17% in another study [23]. Byers et al. reported high to moderate positivity in 20% of the pancreatic cancers analysed [22]. Long non-coding RNA of endogenous retroviral-associated adenocarcinoma (EVADRlncRNA) was expressed moderately to highly in one study, with a mean of 12.1 ± 17.2 reads per kilobase per million mapped reads (RPKM) [26]. Schmitz-Winnenthal et al. reported positivity for HERV-K-MEL in 23% of pancreatic adenocarcinoma samples [16].

### Correlations with immune variables

High ERV load, defined as metastatic samples having an aggregate abundance of transcripts containing endogenous retrovirus greater than the mean ERV load for their respective technical batch, was correlated with antiviral response scores (rho = 0.58, *p* = 0.0005). High ERV samples were correlated with T cells (*p* = 6.9 × 10^− 3^), neutrophils (*p* = 4.6 × 10^− 5^) and Interferon Signaling (*p* = 2.3 × 10^− 4^) signatures [27]. One study showed that high expression of HERV- K was correlated with the presence of antiHERV-K Env SU antibodies (*p* = 0.028) [9], while another showed a correlation with PD-1, PD-L1 and c-Myc expression in Jurkat T cells after transfection with K102-Env or K102-Env-TM expression vectors [25]. High HHLA2 expression was positively correlated with serum cytokines, including IL-2, TNF-α, and IFN-γ, in pancreatic cancer co-culture supernatants, as well as with T cell proliferation (*p* < 0.001) [18], CD8 + T cell infiltration in a clinical cohort of 122 primary tumors (*p* = 0.006) [24], and M2 macrophage infiltration based on bioinformatic analysis (rho = 0.15, *p* = 0.05) [19].

### Correlations with clinical and demographic variables

Regarding demographical and clinical variables, two studies found that HERV-K env protein was directly correlated with more advanced disease stage, 63.6% in stage II A and 100% in stage III, while tumour marker levels and serum HERV-K vRNA levels were higher in metastatic stages (75%) than in localised disease (40%) (*p* = 0.004) [17]. High HHLA2 expression was associated with fewer positive margins postoperatively (15.5% and 56.3%, *p* = 0.012) and negatively correlated with CA 19 − 9 levels (*p* < 0.01). HHLA2 was found to be prevalent in preneoplastic lesions, including 95% of low-grade Pancreatic Intraepithelial Neoplasia and 70.73% of Intraductal Papillary Mucinous Neoplasm cases [24]. No correlation was reported between HHLA2 levels and patient age, gender or AJCC TNM stage [19, 23].

### Survival-related findings

Six studies reported survival outcomes through OS only [18, 19, 23, 24, 26, 27]. Accordingly, two studies reported that retroviral element expression was associated with worse overall survival. EVADR expression was associated with an increased risk of death (HR = 1.47, *p* = 0.02) [26] and HHLA2 overexpression was similarly associated with poorer survival (HR = 1.256, 95% CI: 1.026–1.485; *p* < 0.008) [19]. Conversely, three analyses reported improved outcomes associated with HHLA2 expression [18, 23, 24]. In one study, weak versus strong HHLA2 expression was associated with worse overall survival (HR = 2.241, 95% CI: 1.351–3.781; *p* = 0.002) [18], whereas another study found that high versus low HHLA2 expression was associated with better survival (HR = 0.471, 95% CI: 0.264–0.839; *p* = 0.011) [24]. Additionally, a cohort of 92 patients showed a significant survival difference favouring high HHLA2 expression (log-rank *p* = 0.015) [23].

## Discussion

Our analysis indicates that available evidence is primarily concentrated on two HERV families, with a stronger emphasis on HERV-H–derived elements. HERV-H is the most expressed in colorectal and gastrointestinal cancers [28]. HERV-H on chromosome 3q26 was described as one of the few elements that retain a full open reading frame and that is significantly upregulated in pancreatic cancer cells undergoing epithelial to mesenchymal transition [29]. Another family featured in this review is the HERV-K family, which is the most recently integrated group of endogenous retroviruses in the human genome [30]. HERV-K has been extensively studied in breast cancer [31], but it is significantly upregulated in pancreatic cancer cells compared to healthy tissues [16].

All but one of the included studies investigated HERV expression directly in pancreatic tumor tissue — either in patient samples (clinical cohorts and case series) or in tumor data deposited in public databases such as TCGA (Gibb 2015, Topham 2020). The remaining study (Gong & Xu, 25) assessed HERV-K102 Env in serum samples and in cell-line cultures.

### Levels of expression

Consistently, it was found that high levels of expression were prevalent in pancreatic cancer tissue, while matched or non-matched normal tissue expressed a very low level or none at all. Nevertheless, Yan et al. identified a relatively higher level of expression in preneoplastic lesions such as pancreatic Intra epithelial Neoplasia (PanIN) (95%, 19/20) or intraductal papillary mucinous neoplasm (iPMN) (70.37%, 29/41) and 77.1% (54/70) in pancreatic cancer [23]. It should be noted however that expression varies with study design and analytical techniques. For example, large TCGA identified 25% to 53% expression of EVADR, an ERV1 class of retroviral elements [26]. In contrast, immunohistochemistry on surgical specimens found 77.17% expression of HHLA 2 [23] and 80% of HERV- K env [17]. Also, the biological relevance of findings seems to be influenced by specimen analyzed.

### Which fragments

At the molecular level, most studies focused on regulatory or protein-coding outputs (LTRs and Env) rather than on full-length proviral structures. Env sequences were examined in 3 studies, envelope proteins in 2, LTR sequences in 6 and LTR-derived proteins (HHLA2/B7-H5) in 5 studies. One study evaluated the EVADR long non-coding RNA and one assessed global endogenous retroviral transcript load, irrespective of family.

The EVADR locus on chromosome 6 encodes a 394-nucleotide lncRNA whose transcription is driven by a MER 8 LTR from the ERV 1 family [26]. HERV-K 102 and K108 proviruses contained intact ORF for the env protein and subtype specific proteins have been identified in circulating blood, with K102-Env being found at 36.63-fold higher levels than in healthy controls [25]. Only Li et al. established the presence of an active retroviral system in pancreatic cancer by identifying oncogenic accessory protein transcripts Rec (437 bp) and NP9 (256 bp) in five different cell lines, virus-like particles in Panc-1 and Panc-2 cells and elevated serum vRNA loads [17]. A novel 794 bp splice variant was also reported, which seems specific to pancreatic cancer, belonging to HERV-K gene and probably possessing oncogenic function [17]. HERV-H sequence expression on Xp22.3, an 822 nucleotide ORF, was established through three distinct methods: Northen Blotting and Hybridation, which identified a transcript of 5.4 kb in cell lines and conventional RT-PCR in multiple gastrointestinal cancer lines, revealing overexpression in 17% (2/12) of pancreatic cancer cases [21].

The investigation into HHLA2 as a molecular target for pancreatic cancer has unfolded a conflicting survival signals where the researcher looking at the same protein. Chen, Yan and Boor et their coworkers found that higher expression levels correlated with improved survival outcomes though they arrived at this conclusion using different IHC thresholds: 5% vs. 25%, comparison with normal tissue e.g. [18, 23, 24].

Zhou et al. study reported an unfavorable prognostic signal combing two approaches. A univariable hazard ratio of 2.241 was derived from a forest plot of TCGA data of 178 pancreatic ductal adenocarcinoma (PDAC) while the results of validation cohort of 62 patients were reported only as a Kaplan–Meier curve (log-rank *p* < 0.005) [19].

### Which methods

HERV expression was assessed using RNA, DNA sequencing and immunohistochemistry (IHC), enabling qualitative, quantitative or semi-quantitative evaluation of viral transcripts and envelope or surface protein expression. The published research utilised a range of study designs from a single method on a specimen to a multimodal approach.

Two studies were primarily high-volume analyses. Schmitz Winnenthal ran semi-quantitative RT-PCR of ten antigens considered of viral origin, including HERV-K-MEL [16]. Topham ran an exclusively bioinformatic whole transcriptomic RNA-seq data analysis [27]. Gong and Xu focused on a library construction to generate monoclonal antibodies for a double antibody sandwich ELISA to detect serum biomarkers [25]. In three studies, the basic method was IHC: two of them used tissue microarrays (TMAs) for HHLA2 detection [23, 24] and one stained frozen specimens [22]. The rest of the studies integrated diverse sets of methods, seeking to link retroviral prevalence to functional oncogenic and immunological roles. The study by Zhou et al. combined large-scale bioinformatics analysis (TCGA/GTEx) with validation through IHC on 62 pancreatic cancer tissue specimens and further through Western blot and RT-qPCR to assess HHLA2 in six paired tissues and cell lines [19]. Li et al. investigated the link between active particles and oncogenic signalling by integrating immunohistochemistry (IHC) and RT-qPCR. They detected HERV-K env expression in 80% of pancreatic cancer biopsies and used reverse transcriptase activity assays to identify virus-like particle production, as well as RNA sequencing (RNA-seq) to demonstrate their role as tumor drivers through activation of the RAS–ERK and AKT signalling pathways [17]. In a study by Chen et al., initial findings obtained through immunohistochemistry (IHC), which established HHLA2 as an independent predictor of improved survival, were further evaluated functionally using in vitro T-lymphocyte assays (ELISA) and in vivo xenograft models in B-NSG mice receiving T-cell transfusions [18]. These experiments demonstrated that HHLA2 attenuates tumor growth by enhancing the antitumor immune response. Two studies that focused on individual genomic loci were also multimodal. One study identified and characterized an HERV-H sequence located on chromosome Xp22.3. Initially, suppressive subtractive hybridization was used for its identification, followed by long-distance PCR and 5′ RACE to map its complete structure. This analysis revealed an 822-nucleotide ORF located immediately downstream of the 5′ LTR and showed, through bisulphite sequencing, that its activation is driven by loss of CpG methylation [21]. In a separate large-scale discovery study investigating the structure of EVADR lncRNA, RT-PCR and luciferase reporter assays in cell lines demonstrated that EVADR is a specific biomarker for adenocarcinoma [26].

The breadth of techniques and sample types suggests that HERV expression is detectable across biological compartments but also implies substantial variability in sensitivity and output.

Reported expression frequencies varied markedly by target and method. For HERV-K env-related products, expression ranged from 23% (MEL transcript) to 38% (K102 protein), 63% (K108 protein), and 80% (HML-2) on biopsies. An ORF within a HERV-H sequence on chromosome Xp22.3 was identified using three methods and was found to be ≥ 3-fold overexpressed in 16.7% of pancreatic cancer cases [21]. In contrast, HHLA2 expression showed higher prevalence, ranging from 20% to approximately 80%, with some studies further stratifying expression as absent, low, or high. Positive rates were higher in preneoplastic lesions than in cancerous tissues, reportedly at 95% in low-grade PanIN lesions vs. 77.17% in invasive PDAC [25]. EVADR lncRNA (MER 48 LTR associated) expression was quantified at 12.1 ± 17.2 RPKM, while studies assessing global HERV load reported elevated expression above the cohort mean in subsets of tumors. These data indicate that expression is highly dependent on the specific HERV element measured.

Associations with immune-related variables were reported in 7 of 11 studies. These included correlations with anti–HERV-K antibodies, CD8⁺ tumor-infiltrating lymphocytes, and cytokine levels (IL-2, TNF-α, IFN-γ), reflecting adaptive immune responses; myeloid cell infiltration (M2 macrophages and neutrophils), immune checkpoint molecules (PD-1/PD-L1), CD28H, and c-Myc expression, indicative of immune evasion mechanisms; and interferon signaling, representing innate antiviral responses. Most studies, particularly those evaluating HHLA2, reported that increased tumor-infiltrating CD8⁺ T-cell levels were correlated with higher protein expression. In one large cohort (*n* = 194), the mean number of CD8⁺ cells per millimeter in tumor specimens was significantly higher in cases with HHLA2 expression than in those without [22]. Similarly, Yan et al. reported a direct correlation between CD8⁺ cell density (per unit area) and circulating CD8⁺ cell counts in the bloodstream [23].

Regarding its influence on the adaptive immune response, functional co-culture studies showed that HHLA2-positive cancer cells induce T-lymphocyte proliferation and the production of proinflammatory cytokines ( IL-2, TNF-α, IFN-γ) [18]. PAC patients exhibit significantly higher serum antibodies against Env SU (*p* = 0.0028) and the accessory protein NP9 (*p* = 0.0012) [17]. Downregulation of the CD28H receptor on T lymphocytes, despite high HHLA2 expression by pancreatic cancer cells, represents a mechanism of tumor immune evasion [18]. Additional mechanisms include myeloid cell infiltration and polarization toward M2-type tumor-associated macrophages (TAMs) [19], while ERV transcript load has been directly correlated with neutrophil infiltration signature [27]. HERV-K02 Env circulating protein leads to upregulation of PD-1, PD-L1 and oncogene c-Myc, known to be promoters of tumor evasion [25]. Retroviral expression correlates with measurable interferon signaling signatures, supporting a “viral mimicry” model where the tumor resembles a virally infected cell [27].

Clinical-pathologic associations were reported in 4 studies and included associations with tumor stage, tumor marker levels, T stage, and surgical margin status, with both positive and negative relationships observed, depending on the HERV element evaluated. The prevalence of HERV-K protein increased as disease progressed, from 63.6%. in stage IIA, to 100% in stage III [17]. Serum HERV-K102-env concentration were significantly higher in patients with distant metastasis [8], and the levels of env vRNA and gag vRNA increase dramatically with disease stage [17]. HERV-K102 and CA 19 − 9 were strongly and directly correlated in PDAC patients [25], while HHLA2 expression was more prevalent in cases with elevated CEA levels [23]. Increased HHLA2 expression was significantly inversely associated with T stage (*p* = 0.042) and positively associated with negative surgical margins [24]. These findings indicate that human endogenous retroviral products are quantitative biomarkers of tumor burden.

Survival outcomes were evaluated in six studies. Adverse associations with OS were reported for MER48 LTR expression (HR 1.47, 95% CI 1.06–2.04) [26] and for high HHLA2 expression in two cohorts (HR 2.241 and HR 1.256) [18, 19]. Conversely, two studies reported favorable associations between HHLA2 expression and survival (HR 0.471; HR 0.66; p-values ~ 0.011). Paradoxically, in the study by Chen et al., strong HHLA2 expression was associated with significantly longer median OS (16.5 months) compared with weak expression (11.5 months) [18]. One study reported no significant survival association with ERV aggregates [27]. Overall, the direction of the prognostic association was not consistent across studies, particularly for HHLA2, where one cohort reported an adverse association with survival while three others reported favorable associations. In addition, it was not always evident whether the reported hazard ratios were derived from univariable or multivariable models, whether they were adjusted for clinicopathologic covariates, whether the survival endpoint definitions were comparable across studies or whether the HRs were extracted directly or reconstructed from reported summaries. The available data also do not allow us to determine whether the favorable prognostic signal observed for HHLA2 is preserved in advanced disease or progressively lost as the tumor evolves through immune-evasion mechanisms such as CD28H downregulation. The survival evidence for HHLA2 should therefore be interpreted as preliminary and conflicting rather than as a coherent prognostic signal.

Several mechanistic interpretations of the role of retroviral elements and HHLA2 in pancreatic cancer have been proposed in the included studies. Because they draw on heterogeneous evidence, ranging from in vitro and in silico observations to observational clinical correlations, they should be regarded as proposed or hypothesis-generating rather than as established processes. They can be grouped under four broad themes: epigenetic dysregulation, viral mimicry, HHLA2 functional duality and immune evasion. The activation of normally silent elements like HERV -H Xp 22.3 locus and MER48 LTR is driven by loss of CpG methylation in their viral promoter, a failure of the host cell’s natural defence mechanism that typically uses DNA methylation to silence integrated foreign DNA [21]. High aggregate loads of endogenous retroviral transcripts in advanced pancreatic cancer trigger interferon signalling through accumulation of viral double-stranded RNA in the cancer cells, mimicking a viral infection. A critical host defence mechanism observed in tumour mimicry is translational attenuation of viral protein [27].

HHLA2 operates through two distinct pathways: (i) co-stimulation, via binding to the CD28H (TMIGD2) receptor on naïve T cells [22], which enhances their proliferation and secretion of antitumor cytokines [18]; and (ii) oncogenesis and immunosuppression, mediated through the EGFR/MAPK/ERK and mTOR/AKT signalling pathways, as well as polarization toward M2-type TAMs [19]. Tumours evade immune attack through mechanisms including attenuation of stimulatory receptors, such as CD28H downregulation [18], and the production of circulating HERV-derived proteins that induce PD-1/PD-L1 and c-Myc expression in T cells [25].

### Study limitations

The limitations of this systematic review are related to the search strategy, the eligibility criteria, the quality of the studies included and the biological and analytical heterogeneity of the available data. First, the literature search was restricted to PubMed and Google Scholar, which may have led to the omission of relevant studies. Additionally, there was potential for language bias since only English-language publications were included. The review included eleven studies, which is a relatively small sample for robust conclusions. Second, no single standardized quality assessment tool (such as the Newcastle-Ottawa Scale) could be uniformly applied because of the extreme diversity in study designs, ranging from in vitro experiments to in silico database analyses so, a brief narrative methodological appraisal was used instead when the tool could not be used. Third, detection rates for retroviral elements varied widely (16.7% to 80%) depending on the biological specimen (tissue, serum or cell lines) and the specific laboratory method used. Fourth, the eligibility criteria allowed inclusion of studies that did not address survival, even though one of the review objectives concerned prognostic outcomes; as a consequence, the prognostic synthesis is necessarily based on a smaller subset of the included evidence. Fifth, the included studies investigated biologically and analytically distinct targets (HERV families, specific loci or LTR elements, derived proteins such as HHLA2 and aggregate ERV transcript load), which represent different levels of measurement and may not be directly comparable across studies. In addition, for the survival associations reported, it was not always possible to determine whether hazard ratios were derived from univariable or multivariable models, whether they were adjusted for clinicopathologic covariates, whether the survival endpoint definitions were comparable across studies or whether the HRs were extracted directly or reconstructed from reported summaries. Therefore, evidence remains heterogeneous regarding clinical and prognostic associations, with conflicting survival results reported for the same targets, such as HHLA2 and any cross-study synthesis should be interpreted with caution. Sixth, one of the included studies (Gong & Xu, 25) assessed HERV expression in serum and in cell-line cultures rather than directly in pancreatic tumor tissue, which limits its direct comparability with the tissue-based evidence but contributes complementary mechanistic and circulating-biomarker information on HERV-K102 Env.

Future directions in this field should focus on expanding the evidence base beyond the eleven identified studies. To advance toward clinical utility, research must transition from descriptive expression profiling to mechanistic investigations. This includes elucidating how retroviral elements modulate the broader tumor microenvironment, potentially involving cancer-associated fibroblasts [32], to better define their roles as biomarkers of antitumor immunity or tumour burden. These findings inform the rational design of HERV-targeted immunotherapies for pancreatic cancer along two main directions. HERV-K Env is a promising candidate for mRNA vaccines and chimeric antigen receptor Chimeric Antigen Receptor-T (CAR T) cell therapy due to its high prevalence in PDAC and restricted expression in normal tissues, with CAR-T cells already demonstrating tumor-specific cytotoxicity in other cancer models [33]. Additionally, HHLA2, with its dual co-stimulatory Killer-cell Immunoglobulin-like Receptor 3DL3 (TMIGD2) and co-inhibitory, CD28H, signaling, is being actively explored as a novel B7-family immune checkpoint amenable both to monoclonal-antibody blockade of KIR3DL3 and to engineered TMIGD2-CAR strategies, which may offer a complementary route in tumors refractory to PD-1/PD-L1 blockade [34].

## Conclusions

This systematic review indicates that endogenous retroviral element expression is detectable in pancreatic cancer and may contribute to shaping the tumour microenvironment. A functional duality has been suggested: HERV-K and EVADR have been associated with increased tumour burden and poorer prognosis in some studies, whereas HHLA2 expression has been associated, in several but not all studies, with increased CD8⁺ T-cell infiltration and a trend toward improved survival. The direction and magnitude of these prognostic associations were not uniform across cohorts and were derived from heterogeneous analyses, so they should be interpreted with caution. Retroviral activation may induce a viral mimicry phenotype, while tumours may counteract immune pressure through escape mechanisms such as CD28H downregulation and checkpoint upregulation. All together, these findings support the potential of HERV-derived products as candidate prognostic biomarkers and as promising, hypothesis-generating targets for future immunotherapeutic strategies in pancreatic cancer.

## Supplementary Information

Supplementary Material 1.

Supplementary Material 2.

Supplementary Material 3.

## Data Availability

All the information we collected and analyzed is available upon request from the corresponding author.

## Abbreviations

HERV: Human Endogenous Retrovirus
ERV: Endogenous Retrovirus
LTR: Long Terminal Repeat
RNA: Ribonucleic Acid
mRNA: Messenger Ribonucleic Acid
vRNA: Viral Ribonucleic Acid
DNA: Deoxyribonucleic Acid
HERV-K: Human Endogenous Retrovirus K
HML-2: Human MMTV-like group 2
HERV-H: Human Endogenous Retrovirus H
HHLA2: HERV–H LTR-associating 2
Env: Envelope protein
SU: Surface Unit (of envelope protein)
Gag: Group-specific antigen
Rec: Regulator of Expression
NP9: Nuclear Protein 9
K102-Env: HERV-K102 Envelope protein
K108-Env: HERV-K108 Envelope protein
PD-1: Programmed Cell Death Protein 1
PD-L1: Programmed Death-Ligand 1
CD8⁺: Cluster of Differentiation 8 positive T lymphocytes
T cell: T lymphocyte
IFN-γ: Interferon gamma
TNF-α: Tumor Necrosis Factor alpha
IL-2: Interleukin 2
c-Myc: Cellular Myelocytomatosis Oncogene
CA19-9: Carbohydrate Antigen 19 − 9
EGFR: Epidermal Growth Factor Receptor
MAPK: Mitogen-Activated Protein Kinase
ERK: Extracellular Signal-Regulated Kinase
MEK: Mitogen-Activated Protein Kinase
AKT: Protein Kinase B
mTOR: Mechanistic Target of Rapamycin
RT-PCR: Reverse Transcription Polymerase Chain Reaction
qRT-PCR: Quantitative Reverse Transcription Polymerase Chain Reaction
qPCR: Quantitative Polymerase Chain Reaction
IHC: Immunohistochemistry
WB: Western Blot
ELISA: Enzyme-Linked Immunosorbent Assay
TEM: Transmission Electron Microscopy
NB: Northern Blot
RNA-Seq: RNA Sequencing
IFS: Immunofluorescence Staining
TCGA: The Cancer Genome Atlas
GTEx: Genotype-Tissue Expression project
5’RACE: Rapid Amplification of cDNA Ends
RPKM: Reads Per Kilobase of transcript per Million mapped reads
HR: Hazard Ratio
Rho: Spearman’s correlation coefficient
PDAC: Pancreatic Ductal Adenocarcinoma
PAC: Pancreatic Adenocarcinoma
OS: Overall Survival
CSS: Cancer-Specific Survival
RFS: Recurrence-Free Survival
Quant: quantitative
Qual: qualitative
