# Supplementary material for: Human endogenous retroviruses as immune-related prognostic biomarkers in pancreatic cancer
Source: BMC Cancer. 2026 Aug 20;26:1003. doi: 10.1186/s12885-026-16373-9 (PMC13491691; doi:10.1186/s12885-026-16373-9)
Supplement: Supplementary file 1 [file 12885_2026_16373_MOESM1_ESM.docx]

Tabel 1. Retrieval Strategy and Search Query Medline

((((((((((( "Pancreatic Neoplasms/blood"[Mesh] OR "Pancreatic Neoplasms/drug therapy"[Mesh] OR "Pancreatic Neoplasms/etiology"[Mesh] OR "Pancreatic Neoplasms/genetics"[Mesh] OR "Pancreatic Neoplasms/immunology"[Mesh] OR "Pancreatic Neoplasms/mortality"[Mesh] OR "Pancreatic Neoplasms/pathology"[Mesh] OR "Pancreatic Neoplasms/physiopathology"[Mesh] OR "Pancreatic Neoplasms/therapy"[Mesh] OR "Pancreatic Neoplasms/ultrastructure"[Mesh] OR "Pancreatic Neoplasms/virology"[Mesh] )) OR ( "Carcinoma/blood"[Mesh] OR "Carcinoma/drug therapy"[Mesh] OR "Carcinoma/etiology"[Mesh] OR "Carcinoma/genetics"[Mesh] OR "Carcinoma/immunology"[Mesh] OR "Carcinoma/mortality"[Mesh] OR "Carcinoma/pathology"[Mesh] OR "Carcinoma/physiopathology"[Mesh] OR "Carcinoma/therapy"[Mesh] OR "Carcinoma/ultrastructure"[Mesh] OR "Carcinoma/virology"[Mesh] )) AND ( "Endogenous Retroviruses/classification"[Mesh] OR "Endogenous Retroviruses/drug effects"[Mesh] OR "Endogenous Retroviruses/genetics"[Mesh] OR "Endogenous Retroviruses/immunology"[Mesh] OR "Endogenous Retroviruses/isolation and purification"[Mesh] OR "Endogenous Retroviruses/pathogenicity"[Mesh] OR "Endogenous Retroviruses/ultrastructure"[Mesh] )) AND "Survival"[Mesh]) OR "Mortality"[Mesh]) OR "Disease-Free Survival"[Mesh]) OR "Survival Rate"[Mesh]) OR "Progression-Free Survival"[Mesh]) OR ( "Cell Survival/drug effects"[Mesh] OR "Cell Survival/genetics"[Mesh] OR "Cell Survival/immunology"[Mesh] OR "Cell Survival/physiology"[Mesh] )) OR ( "Tissue Survival/drug effects"[Mesh] OR "Tissue Survival/genetics"[Mesh] OR "Tissue Survival/immunology"[Mesh] )) OR "Survival Analysis"[Mesh]

((((((((( "Pancreatic Neoplasms/blood"[Mesh] OR "Pancreatic Neoplasms/drug therapy"[Mesh] OR "Pancreatic Neoplasms/etiology"[Mesh] OR "Pancreatic Neoplasms/genetics"[Mesh] OR "Pancreatic Neoplasms/immunology"[Mesh] OR "Pancreatic Neoplasms/mortality"[Mesh] OR "Pancreatic Neoplasms/pathology"[Mesh] OR "Pancreatic Neoplasms/physiopathology"[Mesh] OR "Pancreatic Neoplasms/therapy"[Mesh] OR "Pancreatic Neoplasms/ultrastructure"[Mesh] OR "Pancreatic Neoplasms/virology"[Mesh] )) AND ( "Carcinoma/blood"[Mesh] OR "Carcinoma/drug therapy"[Mesh] OR "Carcinoma/etiology"[Mesh] OR "Carcinoma/genetics"[Mesh] OR "Carcinoma/immunology"[Mesh] OR "Carcinoma/mortality"[Mesh] OR "Carcinoma/pathology"[Mesh] OR "Carcinoma/physiopathology"[Mesh] OR "Carcinoma/therapy"[Mesh] OR "Carcinoma/ultrastructure"[Mesh] OR "Carcinoma/virology"[Mesh] )) AND ( "Endogenous Retroviruses/classification"[Mesh] OR "Endogenous Retroviruses/drug effects"[Mesh] OR "Endogenous Retroviruses/genetics"[Mesh] OR "Endogenous Retroviruses/growth and development"[Mesh] OR "Endogenous Retroviruses/immunology"[Mesh] OR "Endogenous Retroviruses/isolation and purification"[Mesh] OR "Endogenous Retroviruses/pathogenicity"[Mesh] OR "Endogenous Retroviruses/ultrastructure"[Mesh] )) AND "Survival"[Mesh]) AND "Mortality"[Mesh]) AND "mortality" [Subheading]) AND "Disease-Free Survival"[Mesh]) AND "Survival Analysis"[Mesh]) AND ( "Survival Rate"[Mesh] OR "Tissue Survival"[Mesh] OR "Cell Survival"[Mesh] OR "Progression-Free Survival"[Mesh])
