# Supplementary material for: Human endogenous retroviruses as immune-related prognostic biomarkers in pancreatic cancer
Source: BMC Cancer. 2026 Aug 20;26:1003. doi: 10.1186/s12885-026-16373-9 (PMC13491691; doi:10.1186/s12885-026-16373-9)
Supplement: Supplementary file 2 [file 12885_2026_16373_MOESM3_ESM.docx]

# Supplementary File S2 — Quality appraisal of the included studies (Newcastle–Ottawa Scale)

The Newcastle–Ottawa Scale (NOS) was applied for cohort/case-series studies to all observational and clinical studies included in this systematic review.

The NOS evaluates three domains: Selection (maximum 4 stars), Comparability (maximum 2 stars), and Outcome (maximum 3 stars), for a maximum total of 9 stars. Studies were graded as Good (≥7 stars, with ≥3 in Selection, ≥1 in Comparability, and ≥2 in Outcome), Fair (4–6 stars), or Poor (≤3 stars).

For studies whose design did not allow a direct individual-patient survival analysis (e.g., cell-line-only or pure in silico analyses), the Outcome items were marked N/A and the Comparability domain was scored only when an analytical comparison was reported. Two studies that were exclusively in silico (Gibb 2015; Topham 2020) are reported separately with a brief narrative methodological appraisal because the NOS framework is not designed for purely bioinformatic studies.

Appraisal was performed independently by two reviewers and adjudicated by a senior author. The findings of this appraisal inform the interpretation of results in the manuscript and are reflected in the Limitations section.

***Item legend***

| Item | Definition |
| --- | --- |
| S1 | Representativeness of the exposed (HERV-positive) cohort. |
| S2 | Selection of the comparator group (HERV-negative or healthy/normal tissue). |
| S3 | Ascertainment of HERV exposure (validated assay; pre-specified threshold). |
| S4 | Demonstration that the prognostic outcome of interest was not present at baseline. |
| C | Comparability of cohorts on the basis of the design or analysis (★ = controlled for the most important PDAC prognostic factor — usually stage; ★★ = additionally controlled for at least one further factor — e.g., grade, treatment, age) |
| Q1 | Assessment of outcome (independent blind assessment, record linkage, or self-report). |
| Q2 | Follow-up long enough for the outcome to occur. |
| Q3 | Adequacy of follow-up of the cohort (≥80% with complete follow-up, or descriptions allowing assessment). |

***Grade thresholds***

| Grade | Thresholds |
| --- | --- |
| Good | 7–9 stars (with ≥3 in Selection, ≥1 in Comparability, ≥2 in Outcome) |
| Fair | 4–6 stars (with ≥2 in Selection, and ≥2 in Outcome where applicable) |
| Poor | ≤3 stars overall, or major deficits in any of the three domains |

**NOS appraisal — observational and clinical studies (n = 9)**

| **#** | Study | Design | Selection (max 4 ★) | | | | Comp. (max 2 ★) | Outcome (max 3 ★) | | | Total ★ | Grade | Notes / rationale |
| --- | --- | --- | --- | --- | --- | --- | --- | --- | --- | --- | --- | --- | --- |
|  |  |  | S1 | S2 | S3 | S4 |  | O1 | O2 | O3 |  |  |  |
| 1 | Schmitz-Winnenthal et al., 2007 (16) | Cell lines + clinical cohort (n=130 PC samples; 23 controls) | ★ | ★ | ★ |  |  | ★ | N/A | N/A | 4/9 | Fair | No survival follow-up; selection of non-exposed (donor + chronic pancreatitis) provides limited comparability adjustment. |
| 2 | Wentzensen et al., 2007 (21) | Case series (n=2/12 PC) |  |  | **★** |  |  | **★** | N/A | N/A | 2/9 | Poor | Very small PC sample; methodological strength in molecular characterisation but minimal clinical inferential value. |
| 3 | Byers et al., 2015 (22) | Case series + cell lines (n=15/23) | ★ |  | ★ |  |  | **★** | N/A | N/A | 3/9 | Poor | Mixed pancreatic and ampullary tumours; limited clinicopathologic adjustment; no survival analysis. |
| 4 | Li et al. (17) | Cell lines + tissue (n=30/30) + serum (n=106 vs 40) | ★ | ★ | ★ |  | ★ | ★ | N/A | N/A | 5/9 | Fair | Comparison with normal pancreatic cell lines and donor sera; multimodal assays; no individual-patient survival analysis. |
| 5 | Chen et al., 2019 (18) | Clinical cohort + in vitro + in vivo (n=136) | **★** | **★** | **★** | **★** | **★★** | **★** | **★** | **★** | 9/9 | Good | Consecutively enrolled (Jan 2012–Feb 2017); multivariable adjustment for TNM, location, chemotherapy; full follow-up |
| 6 | Yan et al. (23) | Clinical cohort with TMA (n=92/153) | **★** | **★** | **★** | **★** | **★** | **★** | **★** |  | 7/9 | Good | Includes PanIN/IPMN comparators; HHLA2 + immune correlates; partial multivariable adjustment. |
| 7 | Boor et al. (24) | Clinical cohort (n=122/194) | **★** |  | **★** | **★** | **★★** | **★** | **★** | **★** | 8/9 | Good | Multivariable model adjusted for clinicopathological factors; pancreatic + ampullary subgroup; complete follow-up |
| 8 | Zhou et al., 2024 (19) | Public DB + clinical (n=345 IHC; 62 paired) | **★** | **★** | **★** |  | **★** | **★** | **★** | **★** | 7/9 | Good | TCGA + GTEx + own validation cohort; orthogonal IHC/RT-qPCR/WB validation; multivariable HR (2.241). |
| 9 | Gong & Xu (25) | Clinical + serum cohort (n=100 PC / 400 controls) | **★** | **★** | **★** |  | **★** | **★** | N/A | N/A | 5/9 | Fair | Healthy and other-cancer controls; orthogonal validation of ELISA limited; no individual survival follow-up. |

**Narrative methodological appraisal — purely in silico studies (n = 2)**

| **#** | Study | Design | Methodological appraisal |
| --- | --- | --- | --- |
| **1** | Gibb et al., 2015 (26) | In silico (TCGA, ENCODE; n=52 PC / 7677) | TCGA-derived analysis with standardised inclusion criteria. RNA-seq quantification with luciferase reporter validation. Univariable HR (1.47, 95% CI 1.06–2.04) for OS; no PC-specific multivariable adjustment for stage or treatment. Methodological strength: high. Inferential limitation: prognostic estimate is unadjusted for known PDAC prognostic factors. |
| **2** | Topham et al., 2020 (27) | In silico, multi-cancer integrated cohort (n=199; 33 PDAC) | Pan-cancer bioinformatic analysis of metastatic tumour cohorts. Standardised RNA-seq quantification of ERV transcript levels with autonomous antiviral signature scoring. Survival analyses pooled across breast, colorectal, and pancreatic adenocarcinoma; no PC-specific multivariable model. Methodological strength: high for the pan-cancer pooled analysis; low for PC-specific prognostic inference because of small subgroup size and absence of stratified survival modelling**.** |

**Summary.** Of the nine NOS-applicable studies, four were graded Good (Chen 2019, Yan, Boor, Zhou 2024), three Fair (Schmitz-Winnenthal 2007, Li, Gong & Xu), and two Poor (Wentzensen 2007, Byers 2015). Most concern arose from the Outcome and Comparability domains, where multivariable adjustment for established PDAC prognostic factors was uneven. Of the two narratively appraised in silico studies, both were judged methodologically strong for the original bioinformatic question but limited in their direct prognostic inferential value for PDAC because of unadjusted survival models or pooled multi-cancer analyses.
